# Elucidating the Effects of Curcumin against Influenza Using In Silico and In Vitro Approaches

**DOI:** 10.3390/ph14090880

**Published:** 2021-08-30

**Authors:** Minjee Kim, Hanul Choi, Sumin Kim, Lin Woo Kang, Young Bong Kim

**Affiliations:** 1 Department of Biomedical Science and Engineering, Konkuk University, Seoul 05029, Korea; mj0411@konkuk.ac.kr (M.K.); chlgksmf9977@hanmail.net (H.C.); 2 Department of Biological Sciences, Konkuk University, Seoul 05029, Korea; fly0000@naver.com (S.K.); lkang@konkuk.ac.kr (L.W.K.)

**Keywords:** curcumin, influenza, inflammation, network pharmacology, molecular docking, systems biology

## Abstract

The influenza virus is a constantly evolving pathogen that challenges medical and public health systems. Traditionally, curcumin has been used to treat airway inflammatory diseases, such as bronchitis and pneumonia. To elucidate common targets of curcumin and influenza infection and underlying mechanisms, we employed network pharmacology and molecular docking approaches and confirmed results using in vitro experiments. Biological targets of curcumin and influenza were collected, and potential targets were identified by constructing compound–disease target (C-D) and protein–protein interaction (PPI) networks. The ligand–target interaction was determined using the molecular docking method, and in vitro antiviral experiments and target confirmation were conducted to evaluate curcumin’s effects on influenza. Our network and pathway analyses implicated the four targets of AKT1, RELA, MAPK1, and TP53 that could be involved in the inhibitory effects of curcumin on influenza. The binding energy calculations of each ligand–target interaction in the molecular docking showed that curcumin bound to AKT1 with the highest affinity among the four targets. In vitro experiments, in which influenza virus-infected MDCK cells were pre-, co-, or post-treated with curcumin, confirmed curcumin’s prophylactic and therapeutic effects. Influenza virus induction increased the level of mRNA expression of AKT in MDCK cells, and the level was attenuated by curcumin treatment. Collectively, our findings identified potential targets of curcumin against influenza and suggest curcumin as a potential therapy for influenza infection.

## 1. Introduction

Seasonal influenza A virus (IAV) is an infectious, enveloped, negative-sense, single-strand RNA virus that is the most common cause of pneumonia-related deaths [1]. Initially, a mild respiratory infection targets the upper respiratory tract, characterized by fever, cough, muscle pain, and fatigue, IAV can lead to severe lethal pneumonia owing to secondary bacterial infection of the lower respiratory tract [2]. Frequent updates of influenza vaccines are necessary, owing to mutations in influenza virus glycoproteins that enable evasion of antibody-mediated immunity induced by vaccination [3]. Together with vaccines, antiviral drugs are used for both treating patients and preventing infection in individuals who have been exposed [2]. However, the emergence of drug-resistant viruses is a major challenge in using antiviral drugs; thus, it is still necessary to explore alternative therapeutic approaches for treating influenza infection [4]. 

Curcumin has attracted considerable research interest because of its versatile pharmacological properties, including anti-inflammatory, antiviral, and anticancer effects [3]. Previous studies have reported that curcumin inhibits the influenza virus by interfering with cellular signaling pathways, including Toll-like receptor and nuclear factor-κB pathways, and disrupting the viral envelope and liposomal membranes [5,6]. However, therapeutic targets and underlying mechanisms of curcumin against influenza have not been fully studied.

To identify and predict targets and underlying mechanisms of curcumin against influenza infection, we implemented a computational research approach and further validated in silico results in vitro. Computational strategies are promising and time-saving alternatives to experimental research that facilitate the identification of targets and disease pathways [7,8]. Herein, we investigated targets associated with curcumin and influenza to determine targets in common, which were further studied through protein–protein interaction (PPI) networks to explore the relationship between the targets. We confirmed biological signaling pathways through the Kyoto Encyclopedia of Genes and Genomes (KEGG) based on common targets. Two networks, compound–disease target (C-D) network and disease target–pathway network (D-P), were constructed and merged to yield a compound–disease target–biological signaling pathway (C-D-P) network to understand underlying mechanisms of curcumin and influenza. A molecular docking study was conducted to evaluate ligand–target interactions between curcumin and proposed targets. Lastly, we performed in vitro antiviral assays to determine antiviral effects of curcumin and target confirmation to observe the underlying mechanism of curcumin against influenza infection. Collectively, our in silico and in vitro approaches identified potent targets and underlying mechanisms of curcumin associated with influenza and established antiviral effects of curcumin. 

## 2. Results

### 2.1. Biological Targets of Curcumin and Influenza

**Figure 1:**
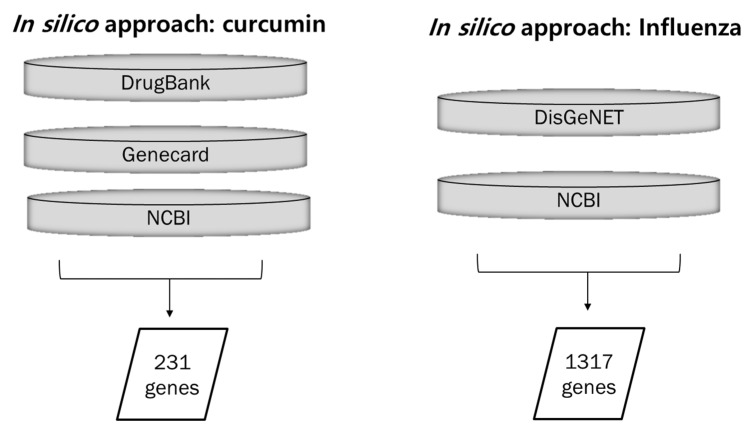
Collection of data for curcumin-, influenza-, and lung inflammation-associated genes. Curcumin-associated genes were collected from three databases: DrugBank, GeneCards, and NCBI. Influenza- and lung inflammation-associated genes were retrieved from two databases: DisGeNET and NCBI.

Overall, 231 *H. sapiens* target genes associated with curcumin were retrieved from DrugBank, GeneCards, and NCBI databases, and 1317 targets associated with influenza virus were identified from DisGeNET and NCBI databases (Figure 1).

### 2.2. Compound–Disease Target Network

**Figure 2:**
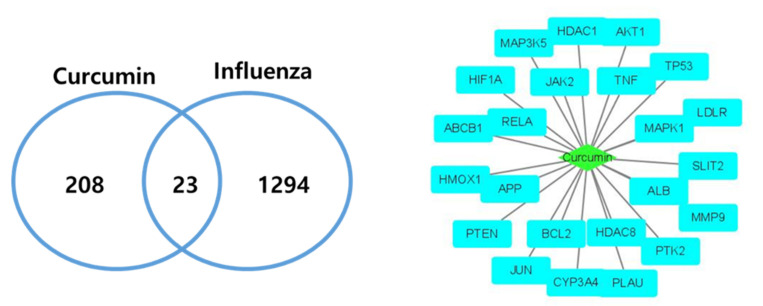
Curcumin–Influenza-associated targets. In total, 23 curcumin and influenza infection common targets were retrieved and constructed as a network.

Among 231 curcumin and 1317 influenza target genes, 23 targets were retrieved as overlapping genes and constructed as a network using Cytoscape (Figure 2). 

### 2.3. KEGG Pathway Analysis 

A KEGG enrichment analysis was further performed on the 23 retrieved curcumin–influenza targets using DAVID bioinformatics resources. The top 20 enriched pathways and diseases (*p* < 0.05) based on gene counts were obtained. This analysis revealed that these targets were enriched for seven viral diseases (hepatitis B, toxoplasmosis, Epstein–Barr virus infection, hepatitis C, influenza A, tuberculosis, and viral carcinogenesis), three inflammation-associated pathways (PI3K-AKT, TNF, and MAPK signaling pathways), and cancer (Table 1).

### 2.4. Protein–Protein Interactions of Disease Targets Associated with Curcumin

**Figure 3:**
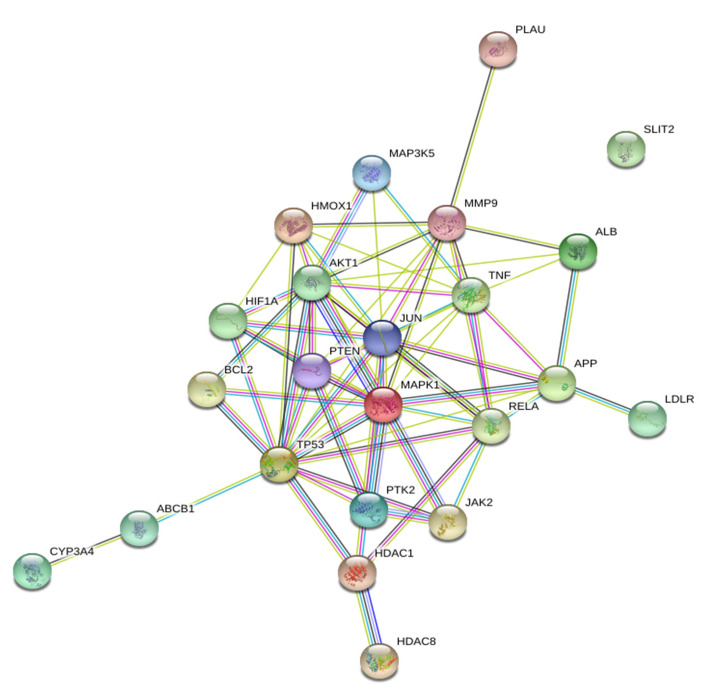
Protein–protein Interaction (PPIs) network constructed using the STRING database. In total, 23 overlapping targets of curcumin and influenza were used as input for the STRING database to construct a PPI network. Two main targets, TP53 (tumor protein 53) and AKT1 (RAC-alpha serine/threonine-protein kinase) were identified in this network.

A protein–protein interaction (PPI) network was constructed with the STRING database using the 23 target genes as input (Figure 3). The database STRING is a global database for the analysis of functional links between proteins. The genes that encode proteins are required for the same function are often located in close proximity to the genome. The graphical representation of the network protein interactions provides a functional linkage, facilitating the analysis of modularity in biological processes [9]. The resulting network revealed two main targets: TP53 (tumor protein 53), which was directly connected to 36 nearest neighbors (degree 36), followed by AKT1 (RAC-alpha serine/threonine-protein kinase: degree 29).

### 2.5. Compound-Disease Target-Pathway (C-D-P) Network

**Figure 4:**
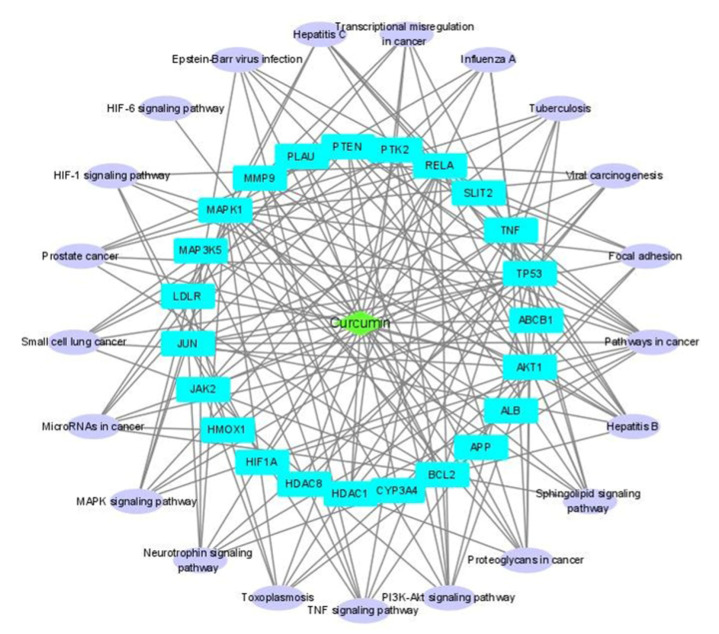
Compound–disease target–pathway (C-D-P) network. C-D network and D-P network were constructed and merged to produce the C-D-P network using Cytoscape.

Two networks, compound–disease target (C-D) network and disease target–pathway network (D-P) were constructed and merged to yield a C-D-P network (Figure 4). The C-D-P network contains 45 nodes and 162 edges. The green diamond in the center, sky blue rectangles, and light purple oblongs correspond to compound, disease targets, and pathways, respectively. Our network analysis showed that AKT1 and RELA (transcription factor p65) exhibited the highest connectedness (degree 18), followed by mitogen-activated protein kinase 1 (MAPK1) (degree 17) and TP53 (degree 15).

### 2.6. Molecular Docking Analysis 

From our two network results (Figure 3 and Figure 4), we selected four proteins—AKT1, TP53, MAPK1, and RELA—as potential targets for curcumin against influenza-associated inflammation. A ligand–target docking approach was used to study the binding affinity (kcal/mol) between curcumin and the targets. The following structures of curcumin and targets were used in the molecular docking: curcumin (the keto form, PDB ID: 6HDR and the enol form, 4PMF); AKT-1 (3O96) [10]; TP53 (3LH0) [11]; MAPK1 (2OJJ) [12]; RELA (1NFI) [13]. The overall structures of the selected target are well conserved with those of the same proteins in different PDBs (Supplementary Table S1). Curcumin exists in two tautomeric forms (keto and enol) [14]; thus, we investigated both forms with the identified targets. The calculated binding affinity (kcal/mol) of each ligand–target identified that curcumin and AKT1 show the strongest binding affinity among all the ligand–target pairs (Table 2).

For the molecular docking, we selected structures in complex with a small molecule (ligand) as the representative structure of each target. The bound ligand was extracted from the original structure and redocked to the same macromolecule. The RMSD value between the structures of original and redocked ligands was measured and used for the validation of the docking protocol. The docking using AutoDock Vina successfully identified the original binding conformations of the ligands in all targets except RELA (Supplementary Table S2). There is no small-molecule ligand-bound structure of RELA. All the RMSD values (Å) of the redocked original ligand from the crystallographic structures were 0.000, which were listed as top solutions in the Supplementary Table S2. In our study, the lowest predicted binding affinity that had RMSD values of zero was selected.

**Figure 5:**
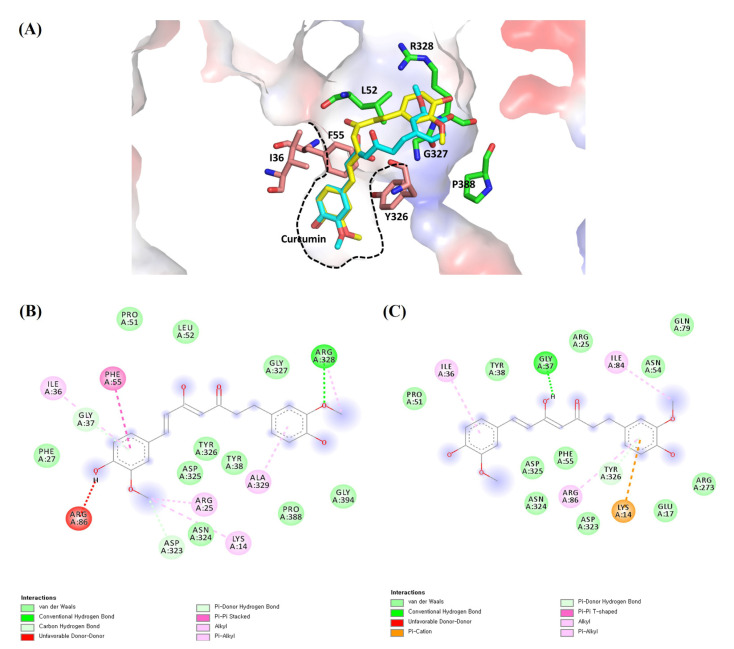
The proposed ligand–receptor interactions of curcumin–AKT1 from molecular docking: (**A**) ligand–receptor interactions of the keto (cyan) and enol (yellow) forms of curcumin with AKT1 showing the electrostatic surface in the 3D representation. Half of the curcumin is tightly bound in AKT1 with the hydrophobic residues (pink) of Ile36, Phe55, and Y326, and the other half is partially exposed to solvent and interacts with the surface residues (green) of Leu52, Gly327, Arg328, and Pro388. The tight hydrophobic pocket is shown in the dashed black line; (**B**) the detailed molecular interactions of the keto form of curcumin with AKT1 in the 2D representation; (**C**) the detailed molecular interactions of the enol form of curcumin with AKT1 in the 2D representation.

Ligand–receptor interactions were identified using Discovery Studio visualizer and verified in Pymol (Figure 5). Curcumin–AKT1 interactions were further analyzed with both the keto (cyan) and enol (yellow) forms of curcumin. Curcumin is a symmetric molecule. In the docked model, half of the curcumin is tightly bound in AKT1, and the other half is partially exposed to solvent (Figure 5A). The internally bound half of curcumin has extensive hydrophobic interactions with Ile36, Phe55, and Tyr326. The other solvent-exposed half of curcumin has the van der Waals interactions with Leu52, Gly327, Arg328, and Pro388. Both keto and enol forms of curcumin show the conserved binding conformation except the rotation of the main chain having the different keto and enol motifs. The curcumin (keto)–AKT1 interaction involves three hydrogen bonds with the key residues Gly37, Asp323, and Arg328, and the van der Waals interactions with Lys14, Arg25, Arg86, Arg328, and Ala329 (Figure 5B). The curcumin (enol)–AKT1 interaction formed two hydrogen bonds with the key residues Gly37 and Tyr326, and the van der Waals interactions with Lys14, Arg86, Ile36, and Ile84 (Figure 5C).

In AKT1, the docking result of the two forms of curcumin was compared to that of the original ligand, AKT1 inhibitor (IQO), in the selected AKT1 structure (PDB ID: 3O96) (Table 3) [10]. The AKT1 inhibitor shows a very low binding energy of <−14.0 Kcal/mol and curcumin also shows reasonable binding energy of <−9.0 Kcal/mol, compared to the previously published docking study [15]. 

### 2.7. Concentration-Dependent Cytotoxic Effects of Curcumin and Oseltamivir against Influenza Virus 

MDCK cells were treated with curcumin and oseltamivir (positive control) for 48 h and stained with crystal violet, after which optical density (OD) was measured at 575 nm using a spectrophotometer. The calculated CC_50_ values of curcumin and oseltamivir were 61.27 and 310.2 µM, respectively (Table 4). 

**Figure 6:**
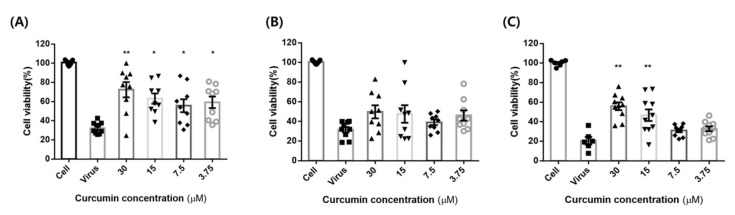
Curcumin cytopathic effect (CPE) test. MDCK cells were treated with influenza virus (100TCID50) with or without different concentrations of curcumin (3.125–30 µM): (**A**) pre-treatment: MDCK cells were pre-treated with different concentrations of curcumin for 1 h at 37 °C and then infected with influenza virus for 48 h to determine curcumin’s prophylactic effect; (**B**) co-treatment: MDCK cells infected with virus were co-treated with different concentrations of curcumin for 48 h at 37 °C to determine curcumin’s virus binding-inhibitory effect; (**C**) post-treatment: MDCK cells were infected with virus for 1 h and then incubated with curcumin for 48 h at 37 °C to validate whether curcumin exhibits therapeutic effects. ** *p* < 0.001, * *p* < 0.05, compared with the virus group; NS, not significant.

To measure the anti-influenza actions of curcumin in MDCK cells, we assessed its effect on the virus cycle by performing antiviral experiments using three paradigms: pre-treatment, co-treatment, and post-treatment. In antiviral tests, MDCK cells infected with the influenza virus (100TCID_50_) were treated with different concentrations of curcumin (3.125–30 µM) according to the indicated paradigm (Figure 6). The greatest effect was observed with pre-treatment, which increased cell viability to more than 50% at concentrations as low as 3.75 µM. Post-treatment also significantly enhanced cell survival at concentrations of 30 and 15 µM, but co-treatment had no effect at any concentration. Collectively, these experiments confirm that curcumin exerts antiviral effects against the influenza virus when applied as a pre-treatment or post-treatment, suggesting that curcumin may be effective in both prophylactic and therapeutic applications.

### 2.8. Inhibitory Effects of Curcumin on Influenza Virus-Induced AKT mRNA Expression

**Figure 7:**
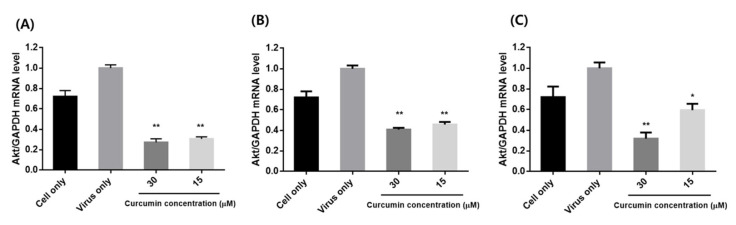
Effects of curcumin treatment on mRNA expression of AKT in influenza-infected MDCK cells: (**A**) pre-treatment: MDCK cells were pre-treated with different concentrations (15 and 30 µM) of curcumin and infected with influenza virus; (**B**) co-treatment: MDCK cells infected with virus were co-treated with different concentrations of curcumin; (**C**) post-treatment: MDCK cells were infected with virus and then incubated with curcumin. ** *p* < 0.001, * *p* < 0.05, compared with the virus group.

The impact of influenza infection on activation of AKT mRNA expression was confirmed by qRT-PCR (Figure 7). The levels of AKT were significantly attenuated by curcumin in all three treatment methods (pre-, co-, and post-treatment). The greatest effect was observed with pre-treatment, which corresponds to our antiviral study. 

## 3. Discussion

In the present study, we conducted a systems biology investigation (in silico), antiviral assay study, and target confirmation (in vitro) of curcumin against the influenza virus. We identified four targets—AKT1, RELA, MAPK1 and TP53—from our C-D-P network analysis, and two targets—TP53 and AKT1—from our PPI network that showed high degrees of interactions in silico. All targets are involved in a wide variety of cellular processes and metabolism. AKT proteins regulate cellular functions including cell proliferation, survival, metabolism, and angiogenesis [16]. RELA encodes the NF-κB subunit, a transcription factor that is initiated by many biological processes such as inflammation, immunity, differentiation, cell growth, tumorigenesis, and apoptosis [17]. MAP kinases are also involved in proliferation, differentiation, transcription regulation, and development [18]. PI3K/AKT and downstream of Toll-like receptors (TLRs), MAPK/NF-κB signaling pathways have been proved to be involved in IAV replication [19,20]. A previous study reported that curcumin significantly suppressed IAV-induced activation of TLR2/4/7, AKT, p38/JNK MAPK, and NF-κB pathways [5]. Another study group reported that curcumin inhibited NF-κB signaling in macrophages, and the production of cytokines/chemokines responding to IAV infection, which corresponds to our result [4]. TP53-encoded proteins respond to diverse cellular stresses to regulate the expression of target genes, thereby inducing cell cycle arrest, apoptosis, senescence, DNA repair, or changes in metabolism [21]. In response to IAV infection, p53 is accumulated and activated, responsible for regulating apoptosis and host antiviral defense [22]. In resting cells, low levels of p53 are maintained due to its interactions with the E3 ubiquitin ligase, MDM2 by continuous proteasomal degradation [22]. IAV infection is reported to weaken the interaction between MDM2 and p53, leading increased level of p53 [22]. Curcumin has been identified as an inhibitor of MDM2 that acts independently of p53 [23]. The inhibitory effects of MDM2 occur at the transcriptional level and appear to involve the PI3K/mammalian target of rapamycin (mTOR)/erythroblastosis virus transcription factor 2 (ETS2) pathway [23]. In this regard, upon curcumin-induced inhibition of MDM2 expression, influenza virus infection may lose its target of interaction, resulting in reduced infection.

Our pathway study identified seven virus-associated diseases, including influenza (tuberculosis, viral carcinogenesis, hepatitis B and C, toxoplasmosis, Epstein–Barr virus infection, and influenza) and three inflammation-associated pathways (PI3K-AKT, TNF, and MAPK signaling pathways). Moreover, our molecular docking study showed that, among the four identified targets, AKT1 showed the highest binding affinity for curcumin, indicating that AKT1 is the most potent target. The calculated binding energy of curcumin to AKT1 was reasonably low as <−9.0 Kcal/mol, indicating a possible inhibitory effect [15]. Lastly, our in vitro study demonstrated that curcumin was effective against influenza virus in both pre- and post-treatment paradigms, and confirmed inhibition of mRNA level of AKT expression suggesting that curcumin could be useful as either a prophylactic or therapeutic influenza agent.

Curcumin, isolated from the rhizome of *Curcuma longa* L. (turmeric), is widely recognized for its anti-inflammatory, antimicrobial, and anticancer effects [24]. Due to its versatile pharmacological properties, curcumin has been considered as another option to replace current influenza therapy [6]. Previous studies reported that curcumin inhibits influenza infection by interfering with multiple cellular signaling pathways, but therapeutic targets and underlying mechanisms of action of curcumin against influenza have not been fully elucidated. Herein, our network study identified three main pathways associated with curcumin and influenza: PI3K/AKT, TNF, and MAPK signaling pathways. Among these pathways, the PI3K/AKT pathway stands out in the context of our identification of AKT1 as a target. PI3K consists of a regulatory subunit (p85) and a catalytic subunit (p110) and is activated by autophosphorylation following binding of the Src homology (SH) domain in p85 to viral proteins in the cytoplasm [25]. Upon activation, PI3K phosphorylates lipid substrates of p110, which serve as second messengers to regulate AKT [26]. AKT plays a central role in modulating cellular pathways such as cell survival, proliferation, and apoptosis [27]. It is also involved in influenza virus uptake and later stages of the virus replication cycle [28]. It has been shown that apoptosis in influenza-infected MDCK cells is AKT dependent and is downregulated by the viral protein NS1, an inducer of host AKT [29]. According to Ehrhardt et al., PI3K targets a very early step of the viral replication cycle [30]. Immunofluorescence microscopy analyses indicated that PI3K is involved in the regulation of virus uptake, as indicated by the accumulation of virus particles at the cell surface upon inhibition of PI3K [30]. Another study group reported that inhibition of the PI3K/AKT pathway in influenza infection leads to a reduction in virus yield, further demonstrating a decrease in viral RNA and protein synthesis that suggested a role for the PI3K/AKT pathway in a late phase of infection, such as virus replication [31]. Overall, these reports support our pathway (PI3K/AKT) and target (AKT1) results for curcumin and influenza, as well as our in vitro study of prophylactic and therapeutic effects of curcumin and AKT target confirmation. It was also reported that curcumin downregulates PI3K/AKT signaling during the lipopolysaccharide (LPS)-induced inflammatory response in microglial cells, indicating that curcumin may inhibit influenza virus infection by regulating the PI3K/AKT pathway and that AKT1 could be a potential target in preventing influenza virus [32]. 

## 4. Materials and Methods

### 4.1. Systems Biology

#### 4.1.1. Pharmacokinetic Evaluation and Therapeutic Targets Associated with Curcumin 

Therapeutic targets of curcumin were obtained from DrugBank (ver.5.1.4, University of Alberta, Alberta, Canada) [33], GeneCards (ver.5.1.4, Weizmann Institute of Science, Rehovot, Israel) [34], and NCBI databases (National Center for Biotechnology Information, U.S. National Library of Medicine, Bethesda, MD, USA) [35]. All searches were performed with “Homo sapiens” selected.

#### 4.1.2. Potential Pathological Target Genes Linked to Influenza and Inflammation 

Influenza infection- and lung inflammation-associated targets were obtained from DisGeNET (ver.7.0, Research Programme on Biomedical Informatics (GRIB), Spain) [36] and NCBI databases [35]. All searches were performed with “Homo sapiens” selected.

#### 4.1.3. Construction of a Compound–Disease Target (C-D) Network

Overlapping genes between curcumin (compound) and disease targets (influenza and lung inflammation) were retrieved and constructed as a network using Cytoscape (ver.3.8, Institute for systems biology, Seattle, WA, USA) [37]. 

#### 4.1.4. Biological Function and Pathway Enrichment Analysis 

Pathways associated with identified target proteins were retrieved from the Database for Annotation, Visualization and Integrated Discovery (DAVID ver. 6.8, National Cancer Institute at Frederick, Frederick, MD, USA) database [38]. Analyses using this database apply statistical approaches to identify significantly enriched or depleted groups of selected genes or proteins. A pathway enrichment analysis was performed using the Kyoto Encyclopedia of Genes and Genomes (KEGG) database (release 99.0, Kyoto University, Kyoto, Japan) [39].

#### 4.1.5. Construction of Protein–Protein Interaction (PPI) Network 

Search Tool for Retrieval of Interacting Genes/Proteins database (STRING ver.11.0, Swiss Institute of Bioinformatics, Basel, Switzerland) [40] was used to collect target and target interactions with confidence scores > 0.7, and the network was analyzed using Cytoscape [37].

#### 4.1.6. Compound–Disease Target–Pathway (C-D-P) Network Construction

Curcumin–disease target and disease target–pathway networks were constructed and visualized using Cytoscape [37]. Pathways with *p*-values < 0.05 were used in this network. The two constructed networks were analyzed and merged to produce a compound–disease target–pathway (C-D-P) network. 

#### 4.1.7. Molecular Docking Analysis

A ligand–target docking approach was used to analyze structural complexes of targets with ligands to understand target specificity. Three-dimensional (3D) structures of targets were downloaded from the RCSB protein data bank (University of California San Diego, La Jolla, CA, USA) [41]. Curcumin exists in two tautomeric forms (keto and enol) [14]. The chemical structures of both forms of curcumin were extracted from X-ray crystal form from the RCSB protein data bank (the keto form, PDB ID: 6HDR [42], and the enol form, PDB ID: 4PMF [43]) using Pymol (ver.2.4.0) [44]. All the structures of targets and ligands were converted to the suitable form of PDB using the Avogadro program (ver.1.90.0, University of Pittsburgh, Pittsburgh, PA, USA) [45] and Discovery Studio [46]. For molecular docking, heteroatoms and waters were deleted, and polar hydrogens were added to the original structure of targets. Docking was carried out with AutoDock Vina (ver.1.1.2, Center of Computational Structural Biology (CCSB)—Scripps Research, CA, USA) [47] using PyRx (ver.0.9.6, Scripps Research, La Jolla, CA, USA) [48] based on scoring functions. The Computer Atlas of Surface Topology of proteins (CASTp) server (ver 3.0, University of Illinois at Chicago, Chicago, IL, USA) [49] was used to locate active site of targets. The 2D- and 3D ligand–target interactions were retrieved using BIOVIA Discovery Studio (ver.4.5, BIOVIA, CA, USA) [46]. To validate the molecular docking, the ligands bound to targets in the selected PDBs were extracted, redocked in the same target structures, and the correlation between the original and redocked structures of ligands was confirmed with the root mean square deviation (RMSD) value. 

### 4.2. In Vitro Experiments

#### 4.2.1. Plant Material and Reagents 

Curcumin was purchased from CSN Pharm (Arlington Heights, IL, USA), and purity (>99%) was confirmed by the manufacturer. Curcumin was dissolved in dimethyl sulfoxide (DMSO) to generate a 10 mM stock solution. Dulbecco’s modified Eagle medium (DMEM) was purchased from HyClone (Logan, UT, USA), and fetal bovine serum (FBS) was obtained from Gibco (Carlsbad, CA, USA). 

#### 4.2.2. Cells and Viruses

The influenza virus, H1N1 (A/Puerto Rico/8/34), was provided by professor Song Chang Sun (College of Veterinary Medicine, Konkuk University, Korea), and Madin-Darby canine kidney (MDCK) cells were purchased from the Korean Cell Line Bank (KCLB, Seoul, Korea). MDCK cells were cultured in DMEM containing 10% FBS and 1% penicillin–streptomycin at 37 °C in a humidified 5% CO_2_ atmosphere. Influenza viruses were cultured in DMEM containing 0.3% BSA, 1 µg/mL trypsin and 1% penicillin–streptomycin at 37 °C and 5% CO_2_. Virus titers were determined using the 50% tissue culture infectious dose (TCID50) according to the Reed and Muench endpoint method.

#### 4.2.3. Detection of Cytotoxicity

MDCK cells were seeded into 96-well plates at a concentration of 1 × 10^4^ cells/well. After 24 h, cells were treated with serial dilutions of curcumin for 48 h 37 °C and then stained with crystal violet. Cell viability at each concentration was determined by measuring the optical density (OD) at 575 nm using a spectrophotometer, and the CC50 value, defined as the concentration that induced 50% cell death, was determined. Oseltamivir was used as a positive control.

#### 4.2.4. Antiviral Assay: Pre-Treatment, Co-Treatment, and Post-Treatment

MDCK cells were seeded in a 96-well plate (1 × 10^4^ cells/well) and, after 24 h, were treated with the indicated concentrations of curcumin and infected with 100TCID50 of influenza virus. Antiviral effects of curcumin were evaluated using three methods: (a) pre-treatment, carried out by preincubation of cells with curcumin for 1 h at 37 °C and then incubation with influenza; (b) co-treatment, performed by incubating influenza and curcumin together for 1 h at 37 °C; (c) post-treatment, conducted by preincubation of cells with influenza for 1 h at 37 °C, followed by incubation with curcumin for 1 h at 37 °C. After incubating for 2 d, cells were stained with crystal violet, and optical density (OD) at 575 nm was measured using a spectrophotometer.

#### 4.2.5. Quantitative Real-Time PCR: Pre-Treatment, Co-Treatment, and Post-Treatment

MDCK cells were seeded in a 6-well plate (5 × 10^5^ cells/well) with curcumin (15 and 30 μM) by pre-, co-, and post-treatment and infected with influenza virus. Total RNA was isolated by RNeasy Mini kit (Qiagen, CA, USA) from MDCK cells, and 1 μg of the total RNA was reverse-transcribed into cDNA with the SuperScript II reverse transcriptase (Invitrogen, Carlsbad, CA, USA). Quantitative real-time PCR (qRT-PCR) was performed with a SsoAdvanced Universal SYBR^®^ Green Supermix kit (Bio-Rad, Hercules, CA, USA). Cycling parameters of the qPCR were as follows: 1 cycle at 95 °C for 3 min, followed by 39 cycles at 95 °C for 10 s, 60 °C for 30 s in the on the LightCycler 96 system (Roche, Mannheim, Germany). Primer sequences were as follows: AKT forward, 5′-CAA GTC CTT GCT TTC AGGGC-3′ and reverse, 5′-ATA CCT GGT GTC AGT CTC CGA-3′ (184-bp product); GAPDH forward, 5′-TGT CCC CAC CCC CAA TGT ATC-3′ and reverse, 5′-CTC CGA TGC CTG CTT CAC TAC CTT-3′ (100-bp product). Expression levels of the AKT gene were normalized to glyceraldehyde-3-phosphate dehydrogenase (GAPDH).

#### 4.2.6. Statistical Analysis 

All data were analyzed using the GraphPad Prism6 software (GraphPad Software Inc., San Diego, CA, USA), and a value of *p* < 0.05 was considered statistically significant. The measurements were assessed by one- or two-way ANOVA.

## 5. Conclusions

In summary, through an in silico study, we identified AKT1 as a potential target and the PI3K/AKT pathway as possibly the main pathway associated with curcumin’s action against influenza. We also demonstrated the prophylactic and therapeutic effects of curcumin in vitro and confirmed AKT inhibition by curcumin treatment indicating curcumin’s potential in treating influenza via AKT inhibition. Given the wide availability of curcumin, there should be no significant barriers to clinical trials, which are warranted to confirm curcumin as a future influenza treatment.

## Figures and Tables

**Table 1:** Top 20 enriched KEGG pathways and diseases.

Pathway and Disease (KEGG)	Gene Counts
hsa05200: Pathways in cancer	11
hsa05161: Hepatitis B	9
hsa04071: Sphingolipid signaling pathway	8
hsa05205: Proteoglycans in cancer	8
hsa04151: PI3K-AKT signaling pathway	8
hsa04668: TNF signaling pathway	7
hsa05145: Toxoplasmosis	7
hsa04722: Neurotrophin signaling pathway	7
hsa04010: MAPK signaling pathway	7
hsa05206: MicroRNAs in cancer	7
hsa05222: Small cell lung cancer	6
hsa05215: Prostate cancer	6
hsa04066: HIF-1 signaling pathway	6
hsa05169: Epstein–Barr virus infection	6
hsa05160: Hepatitis C	6
hsa05202: Transcriptional misregulation in cancer	6
hsa05164: Influenza A	6
hsa05152: Tuberculosis	6
hsa05203: Viral carcinogenesis	6
hsa04510: Focal adhesion	6

PI3K, phosphoinositide 3-phosphate kinase; TNF, tumor necrosis factor: MAPK, mitogen-activated protein kinase; HIF-1, hypoxia-induced factor 1.

**Table 2:** The calculated binding affinity of ligand–target.

Ligand–Target	Binding Affinity (kcal/mol) (KETO)	Binding Affinity (kcal/mol) (ENOL)
CM-AKT1	−9.2	−9.0
CM-TP53	−7.0	−7.1
CM-MAPK1	−7.0	−6.9
CM-RELA	−6.9	−7.0

CM, curcumin, KETO: keto form of curcumin, ENOL: enol form of curcumin.

**Table 3:** Ligand–target binding affinity, compared to AKT inhibitor IQO.

Ligand–Target	Binding Affinity (kcal/mol)
CM(keto)-AKT1	−9.2
CM(enol)-AKT1	−9.0
IQO (AKT inhibitor)-AKT1	−14.4

**Table 4:** Cytotoxic effects of curcumin in MDCK cells.

Compound	CC_50_ (µM)
Curcumin	61.27 ± 1.69
Oseltamivir	310.2 ± 1.55

CC_50_, 50% cytotoxic concentration (*n* = 3).

## Data Availability

Data sharing contains in this article and supplementary materials.

## Supplementary Materials

The following are available online at https://www.mdpi.com/article/10.3390/ph14090880/s1, Table S1: The structural similarity between the selected target structures used in docking and other structures of the same targets in PDB. Table S2: RMSD values between the structures of the original and redocked ligands.
