# Supplementary material for: Elucidating the Effects of Curcumin against Influenza Using In Silico and In Vitro Approaches
Source: Pharmaceuticals (Basel). 2021 Aug 30;14(9):880. doi: 10.3390/ph14090880 (PMC8465221; doi:10.3390/ph14090880)
Supplement: Supplementary file 1 [file pharmaceuticals-14-00880-s001.zip › pharmaceuticals-1296060-SI.pdf]

Supplementary Table S1. The structural similarity between the selected target structures used in docking and other structures of the same targets in PDB.

Target proteins	The total number of amino acids in full-length protein	Selected target structures used in docking	The number of amino acids of selected targets	Other structures of the same targets in PDB	RMSD between selected targets and other structures of the same targets	The number of aligned amino acids in the RMSD calculation
AKT1	480	3O96	367	4GV1	1.181	250
				6HHF	0.793	301
				5KCV	0.751	341
				4EJN	0.761	355
				4EKL	1.099	262
				3QKM	1.106	262
				6S9X	0.893	358
TP53	393	3LH0	120	2G3R	0.165	99
				6MXY	0.63	112
				4CRI	0.390	115
MAPK1	360	2OJJ	344	4ZXT	0.478	338
				5LCJ	0.36	336
				5BVE	0.963	323
				1TVO	0.403	343
				6GDQ	0.228	334
				4NOS	0.426	336

				**3W55**	**0.961**	**331**
				**4QTA**	**0.476**	**315**
				**6G54**	**0.407**	**339**
				**4ZZO**	**0.294**	**336**
**RELA**	**551**	**1NFI**	**295**	**3GUT**	**1.207**	**272**

The selected target proteins are AKT1 (protein ID: AAA36539.1), TP53(XP_011520288.1), MAPK1(NP_002736.3) and RELA(AAA36408.1).

Some amino acids of AKT1 in the structures of 6HHF, 5KCV, 4EJN, 4EKL, 3QKM, and 6S9X are mutated.

Supplementary Table S2. RMSD values between the structures of the original and redocked ligands.

Target protein	Ligand ID	Calculated binding affinity (kcal/mol)	RMSD (Å)	
			upper bound	lower bound
AKT1 (PDB ID: 3O96)	IQO	-14.4	0	0
		-14.1	2.038	1.208
		-13.2	4.87	2.612
TP53 (PDB ID: 3LH0)	PGE	-3.7	0	0
		-3.5	5.573	0.89
		-3.6	19.158	18.286
		-3.3	19.503	18.714
		-3.6	20.72	18.905
		-3.3	20.761	19.858
		-3.5	21.158	20.226
		-3.2	22.298	21.395
		-3.3	29.826	28.866
MAPK1 PDB ID: 2OJJ	82A	-8.1	0	0
		-8.1	2.094	1.726
		-8.1	30.624	27.181
		-7.9	31.093	28.293
		-7.8	20.798	18.637
		-7.6	1.992	1.466

		**-7.6**	**28.769**	**27.233**
		**-7.4**	**10.47**	**6.225**
		**-7.4**	**21.947**	**19.505**

The RMSD/upper bound represents the RMSD value matching each atom in one conformation with the same atom in the other conformation, ignoring any symmetry. The RMSD/lower bound represents the RMSD value matching each atom in one conformation with the closest atom of the same element type in the other conformation [1].

Reference

[1] O. Trott, A.J. Olson, AutoDock Vina: improving the speed and accuracy of docking with a new scoring function, efficient optimization, and multithreading, Journal of computational chemistry 31(2) (2010) 455-61.
